# Account of Diseases in an Eastern District of London, from the 20th of April to the 20th of May, 1800

**Published:** 1800-06

**Authors:** 


### STATE OF DISEASES IN LONDON.

*Account of Diſeaſes in an Eaſtern Diſtrict of London, from the 20th of April to the 20th of May,* 1800.

| | No. of Caſes. |
|---|---|
| ACUTE DISEASES. | |
| Typhus mitior | 4 |
| Peripneumonia | 2 |
| Pleuritis | 1 |
| Sore Throat | 1 |
| Acute Rheumatiſm | 2 |
| CHRONIC DISEASES. | |
| Cough | 10 |
| Dyſpnœa | 5 |
| Cough with Dyſpnœa | 7 |
| Hæmoptyſis | 5 |
| Phthiſis Pulmonalis | 6 |
| Dyſpepſia | 8 |
| Bilious Vomiting | 2 |
| Gaſtrodynia | 7 |
| Diarrhœa | 14 |
| Enterodynia | 6 |
| Conſtipatio | 3 |
| Colica | 2 |
| Hepatalgia | 1 |
| Aſcites | 3 |
| Hydrothorax | 2 |
| Anaſarca | 4 |
| Apoplexy | 2 |
| Vertigo | 4 |
| Hyſteria | 3 |
| Epilepſia | 1 |
| Paralyſis | 2 |
| Chronic Rheumatiſm | 16 |
| Scrophula | 2 |
| Prurigo | 1 |
| Tinea | 1 |
| PUERPERAL DISEASES. | |
| Inflammatio mammarum | 1 |
| Menorrhagia lochialis | 3 |
| Dolores poſt partum | 5 |
| INFANTILE DISEASES. | |
| Ophthalmia | 2 |
| Tinea | 1 |
| Scrophula | 3 |
| Convulſio | 4 |
| Croup | 1 |

The variety in the ſtate of the weather, which has occurred ſince the laſt report, has been productive of ſome change in the conſtitution, and in the ſtate of diſeaſe. During the few warm days at the beginning of the month, the number of pneumonic complaints was leſſened, or the ſymptoms of them greatly mitigated.

The great degree of warmth, however, gave occaſion, in many inſtances, to a haſty and imprudent change of cloathing; in in conſequence of which, upon the return of eaſterly and north-eaſterly winds, ſome, who were recovering from previous diſeaſe, ſuffered a relapſe, and others were expoſed to catarrhal or rheumatic affections.

To the ſame cauſes alſo may be attributed many complaints of the bowels, which have prevailed for ſome time. Diarrhœas, colic, dyſentery, and cholera are frequently the diſeaſes of autumnal months, which have ſucceeded a very warm ſummer; and ſome ſymptoms of theſe complaints, which have lately prevailed, though in a lower degree, may be attributed to the ſudden variation of temperature. But, whilſt diarrhœa has proved troubleſome in ſome inſtances, a different and oppoſite ſtate of the bowels, viz. an obſtinate conſtipation has occurred in others. This diſeaſe is very apt to prove troubleſome to perſons in advanced life, and not unfrequently is accompanied with ſerious and alarming ſymptoms. It may be traced to a variety of cauſes, and occurs under very different circumſtances of the conſtitution. It is ſometimes the attendant of high health, and the conſequence of ſtrong exerciſe, producing a rigid ſtate of the muſcular fibres; at other times, it occurs to perſons of a different conſtitution, and who are ſubject to different habits. In ſome patients, who are of a weak and relaxed temperament, the bowels, partaking of the general affection of the ſyſtem, act but feebly on their contents, and their evacuation is but ſlowly promoted. Bile, which is the natural cathartic, is defective in its quantity, or is not ſent forward regularly into the inteſtines. This ſtate of the ſyſtem is generally indicated by a peculiar appearance of the countenance, and is frequently accompanied by ſymptoms of hypochondriaſis, and what has been termed nervous affections. Theſe circumſtances occurred in the inſtances referred to in the liſt, and the frequent uſe of different cathartic remedies became neceſſary. Aloes and rhubarb were found uſeful in theſe caſes; but it was ſometimes neceſſary to have recourſe to the more active operation of jalap, cathartic extract, or calomel.

